# 471. Broader Than Necessary: A Qualitative Study Exploring Broad-spectrum Antibiotic Prescribing Tendencies Using A Situated Cognition Educational Framework

**DOI:** 10.1093/ofid/ofaf695.159

**Published:** 2026-01-11

**Authors:** Marc Trubin, Elizabeth A Scruggs-Wodkowski, Emily Abdoler

**Affiliations:** University of Michigan Healthcare System, Ann Arbor, Michigan; VA Ann Arbor Healthcare System, Ann Arbor, Michigan; University of Michigan, Ann Arbor, MI

## Abstract

**Background:**

Effective antibiotic choice is imperative to high quality care. However, antibiotics used in the hospital are often unnecessarily broad-spectrum, leading to increased rates of adverse effects and higher costs to the healthcare system. Prior work has explored how clinicians select antibiotics but not why they use agents that are broader than necessary. Using Situated Cognition – an educational theory positing that knowledge acquisition is inseparable from its setting – as a framework, we sought to improve our understanding of how clinicians conceptualize, justify, and manage broad-spectrum antibiotics in the inpatient setting.

**Figure:**
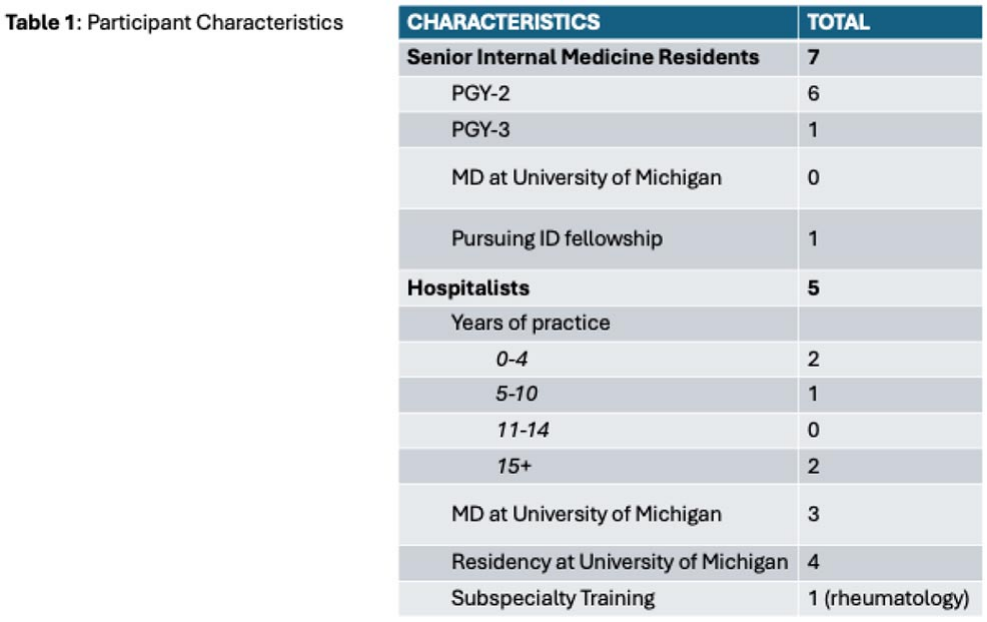

**Figure:**
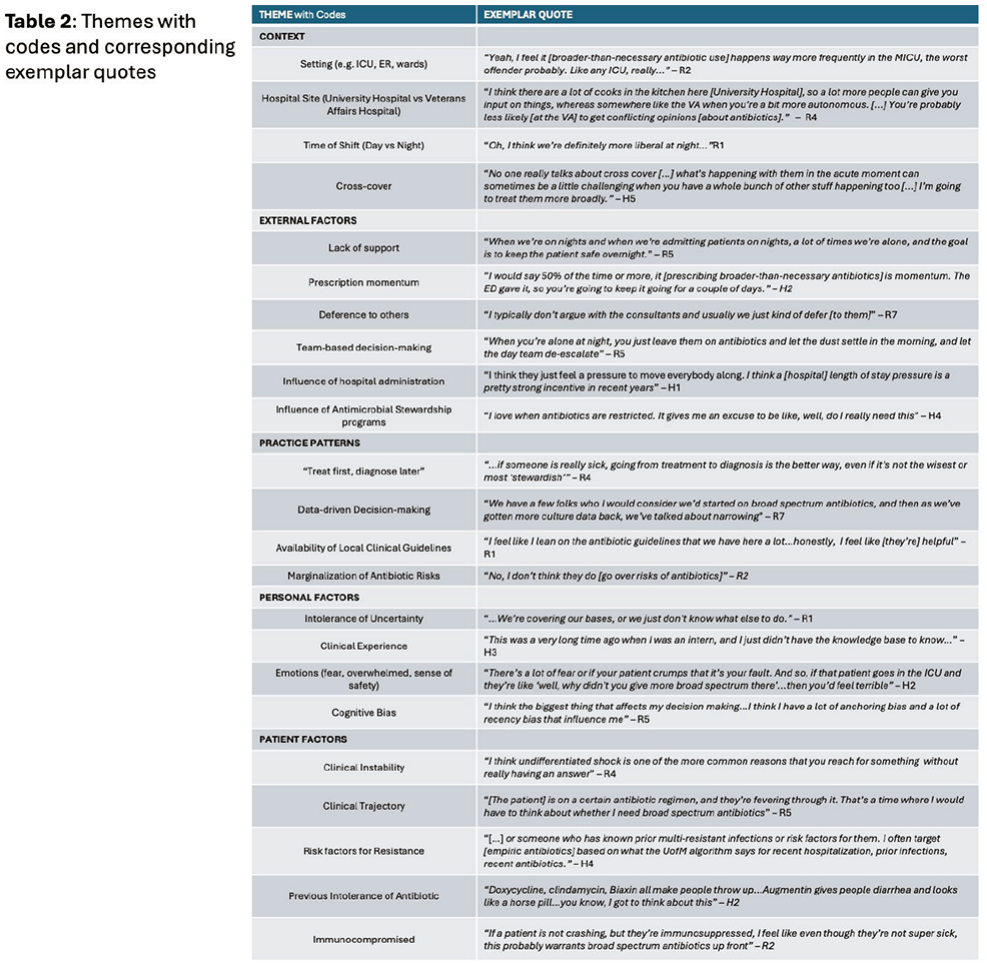

**Methods:**

We conducted 12 semi-structured interviews with hospitalists and senior Internal Medicine residents who practice at the University of Michigan and Ann Arbor Veterans Affairs hospitals (Table 1). Interviews explored participants’ experiences with antibiotics in different inpatient settings. Employing Situated Cognition as a sensitizing framework, we generated a codebook from interview transcripts through an iterative, inductive process. We consolidated codes into themes using Braun & Clark’s thematic analysis approach.

**Figure:**
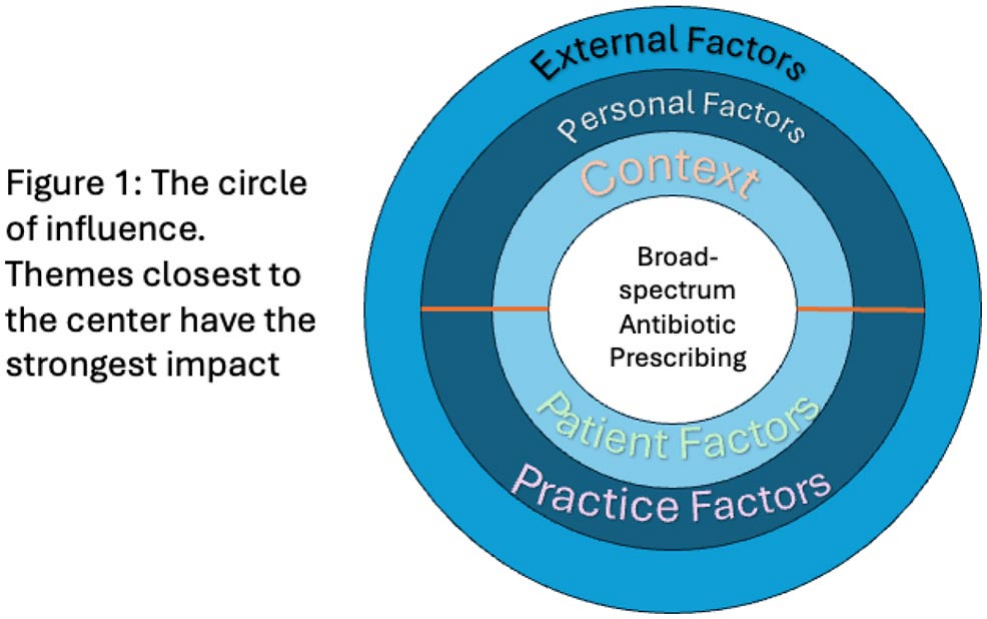

**Results:**

We identified 24 factors impacting antibiotic selection that coalesced into 5 themes: *context*, *external factors*, *practice patterns*, *personal factors*, and *patient factors* (Table 2). Condensing these concepts revealed a framework of influence with *context* and *patient factors* having the most effect and *external factors* the least (Figure 1). Most participants described challenges when selecting antibiotics at night and when cross-covering others’ patients.

**Conclusion:**

Our results build on prior management reasoning research, using the lens of Situated Cognition to better understand broad-spectrum antibiotic use. Situated Cognition effectively describes the interactions between the provider, patient, and the environment as they relate to antibiotic prescribing and illuminates situations with the potential for impactful intervention. Notably, our study identified nighttime and cross-cover as high priority settings with the potential to improve antibiotic decision-making through educational initiatives with the goal of combating unhelpful external pressures.

**Disclosures:**

All Authors: No reported disclosures

